# Supplementary material for: The C. difficile toxin B membrane translocation machinery is an evolutionarily conserved protein delivery apparatus
Source: Nat Commun. 2020 Jan 23;11:432. doi: 10.1038/s41467-020-14306-z (PMC6978384; doi:10.1038/s41467-020-14306-z)
Supplement: Supplementary file 1 — Supplementary Information [file 41467_2020_14306_MOESM1_ESM.pdf]

Supplementary Figures

The *C. difficile* toxin B membrane translocation machinery is an evolutionarily conserved protein delivery apparatus

Kathleen E. Orrell[1,2], Michael J. Mansfield[3], Andrew C. Doxey[3], Roman A. Melnyk[1,2]*

Supplementary Figure 1

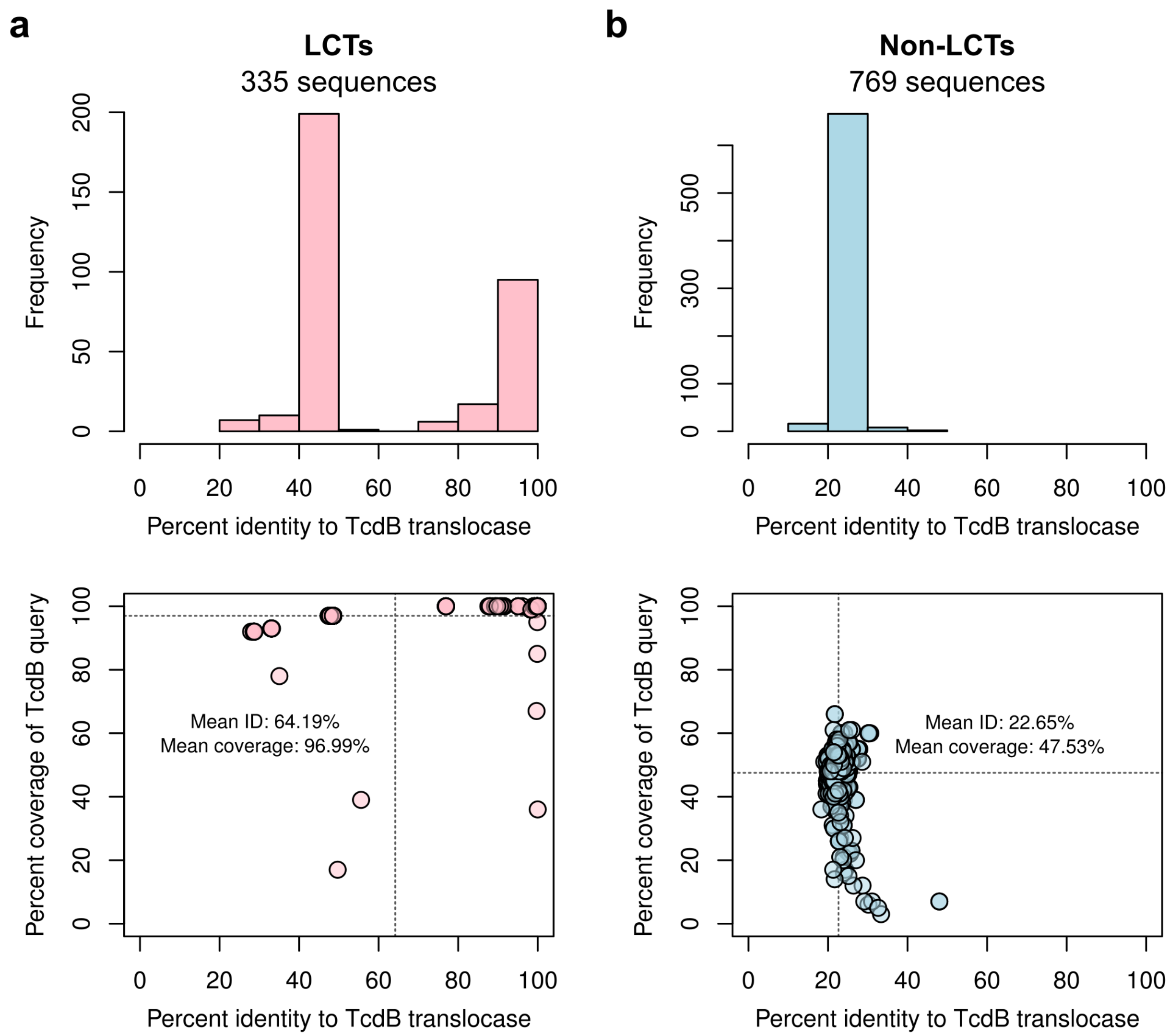

Supplementary Figure 1 Comparison of detected clostridial LCT (**a**) and non-LCT (**b**) translocases to the TcdB translocase. The LCT translocases correspond to partial and complete translocase sequences from TcdA, TcdB, TcsL, TcsH, TpeL, and TcnA proteins, which possess greater sequence identity over a larger portion of the TcdB translocase compared to LCT-like translocases found outside of the clostridia.

Supplementary Figure 2

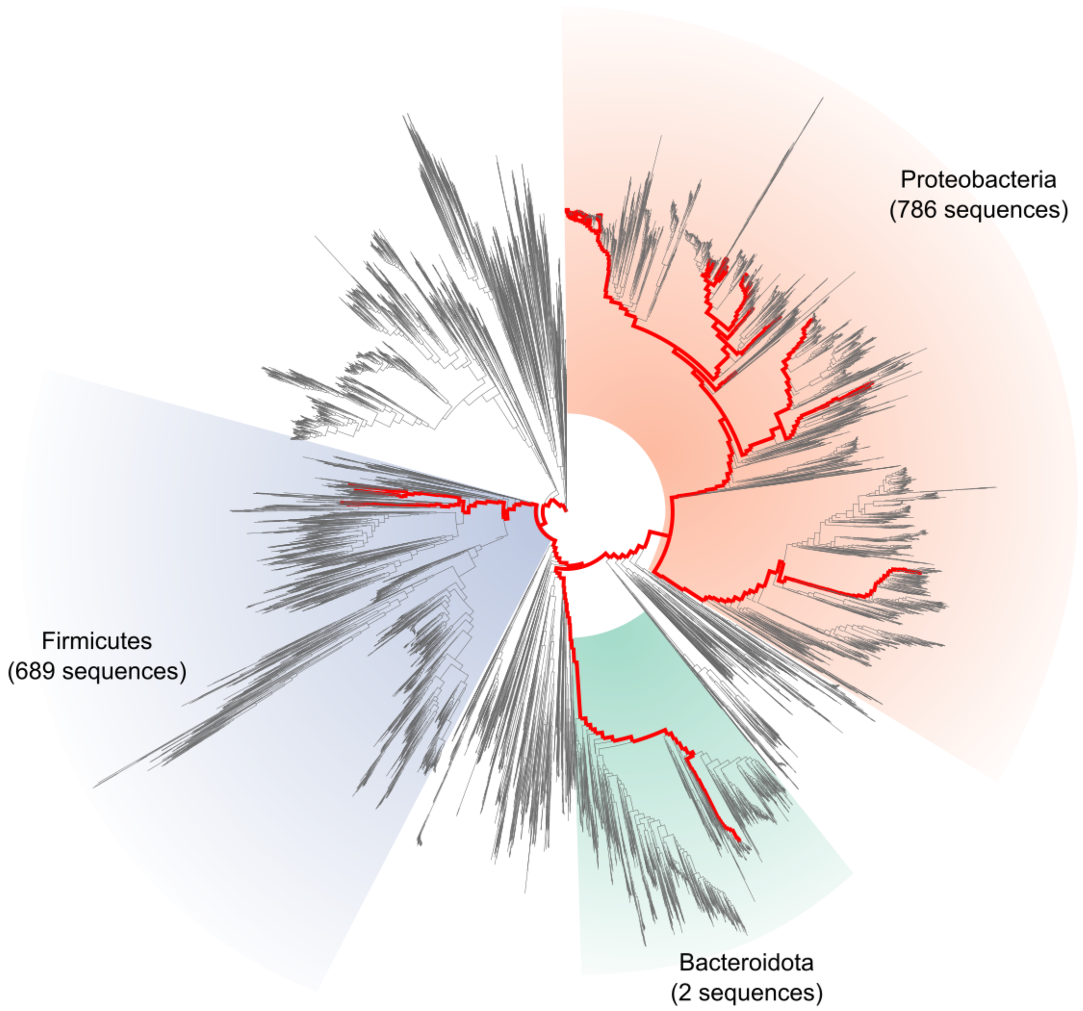

Supplementary Figure 2 Phylogenetic distribution of bacterial species with a PSI-BLAST match to the TcdB T-domain. Lineages that contain an LCT-like T-domain (red highlighting) are found mostly within the Firmicutes and Proteobacteria. Visualization of phylogenomic distributions was performed using AnnoTree[1].

Supplementary Figure 3

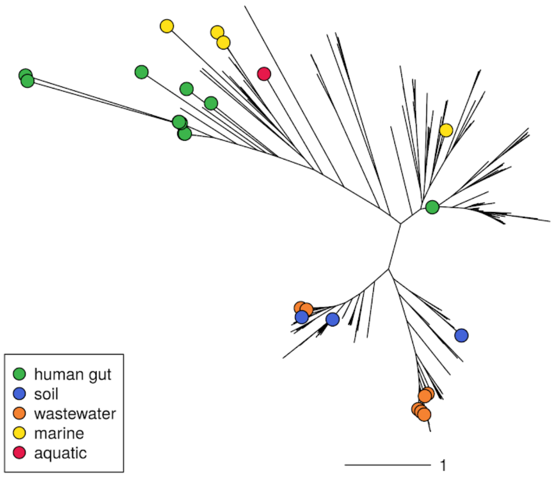

Supplementary Figure 3 Phylogenetic placement of metagenomic homologs on the LCT-T domain phylogeny shown in Fig. 1b.

Supplementary Figure 4

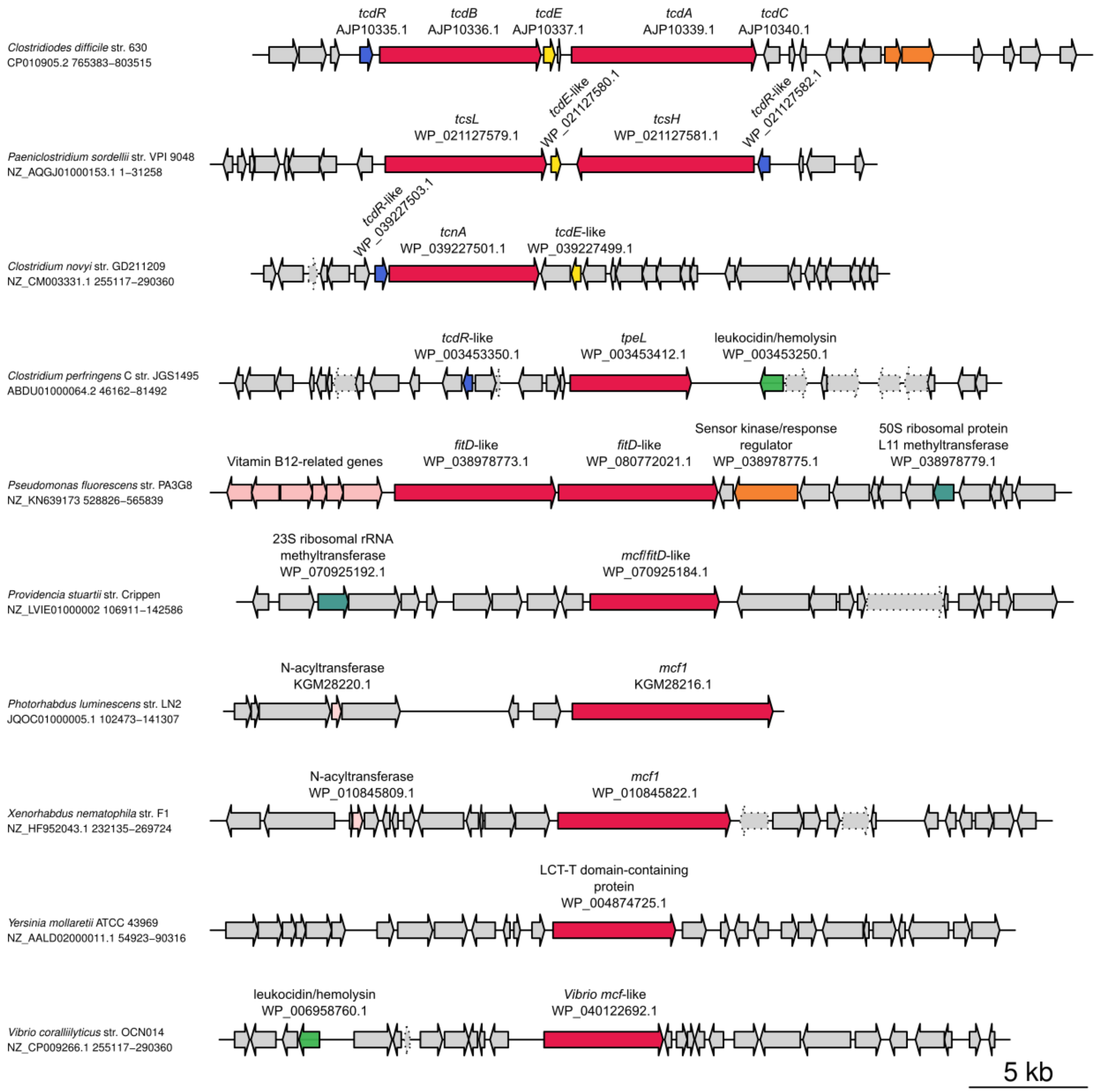

Supplementary Figure 4 Genomic context from a representative set of LCT-like translocation domain-containing genomes. The contents extracted include $\pm$ 15-kb upstream and downstream of the LCT gene boundaries (mean length 34.7-kb $\pm$ 5.6-kb)In *C. difficile*, the LCT genes *tcdA* and *tcdB* are found in a pathogenicity locus (PaLoc) that also contains the regulator *tcdR* and the holin gene *tcdE*, responsible for exporting the toxin genes. Other LCTs are found in loci with some shared features. Outside of the clostridia, proteins with LCT-like translocation domains are found in a wider variety of genomic contexts. These proteins are associated with the presence of secretion systems, but otherwise have few commonalities. One apparently conserved locus, shared between many species of *Pseudomonas*, is related to B12 metabolism.

Supplementary Figure 5

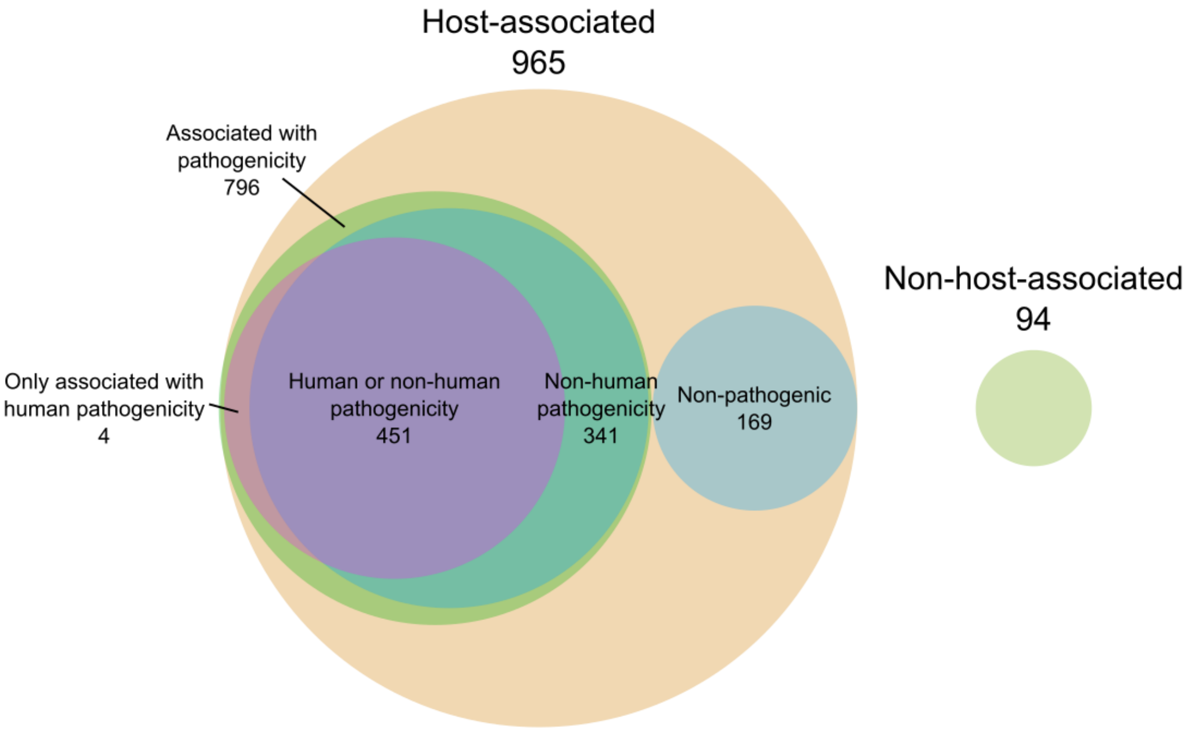

Supplementary Figure 5 Associated pathogenicity analysis of species containing LCT-T proteins. (**a**) Of 247 unique species represented in the dataset, 189 species (representing 91% of all sequences) were associated with a host, suggesting the presence of LCT-T homologs correlates strongly with host association. Of these host-associated species, 105 species (representing 82% of host-associated sequences) were associated with pathogenicity, with 20 species associated with known human pathogenicity (representing 57% of pathogen-associated sequences). Source data are provided as a Source Data File.

Supplementary Figure 6

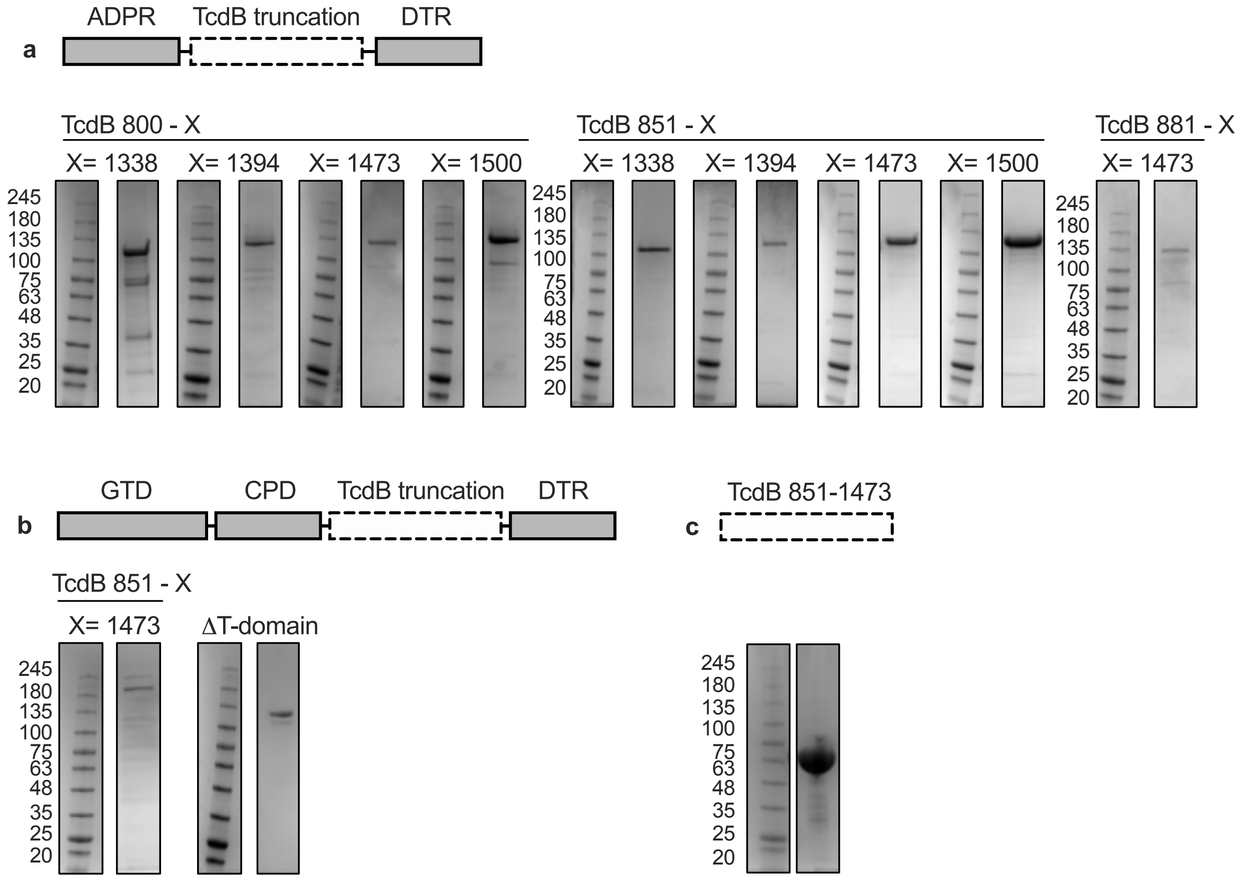

Supplementary Figure 6 SDS-PAGE of purified constructs. (**a**) ADPR-[truncated TcdB T-domain]-DTR chimeras, with the truncated T-domain indicated in the figure. (**b**) GTD-CPD-TcdB(851-1473)-DTR and ΔT-domain. (**c**) TcdB 851-1473.

Supplementary Figure 7

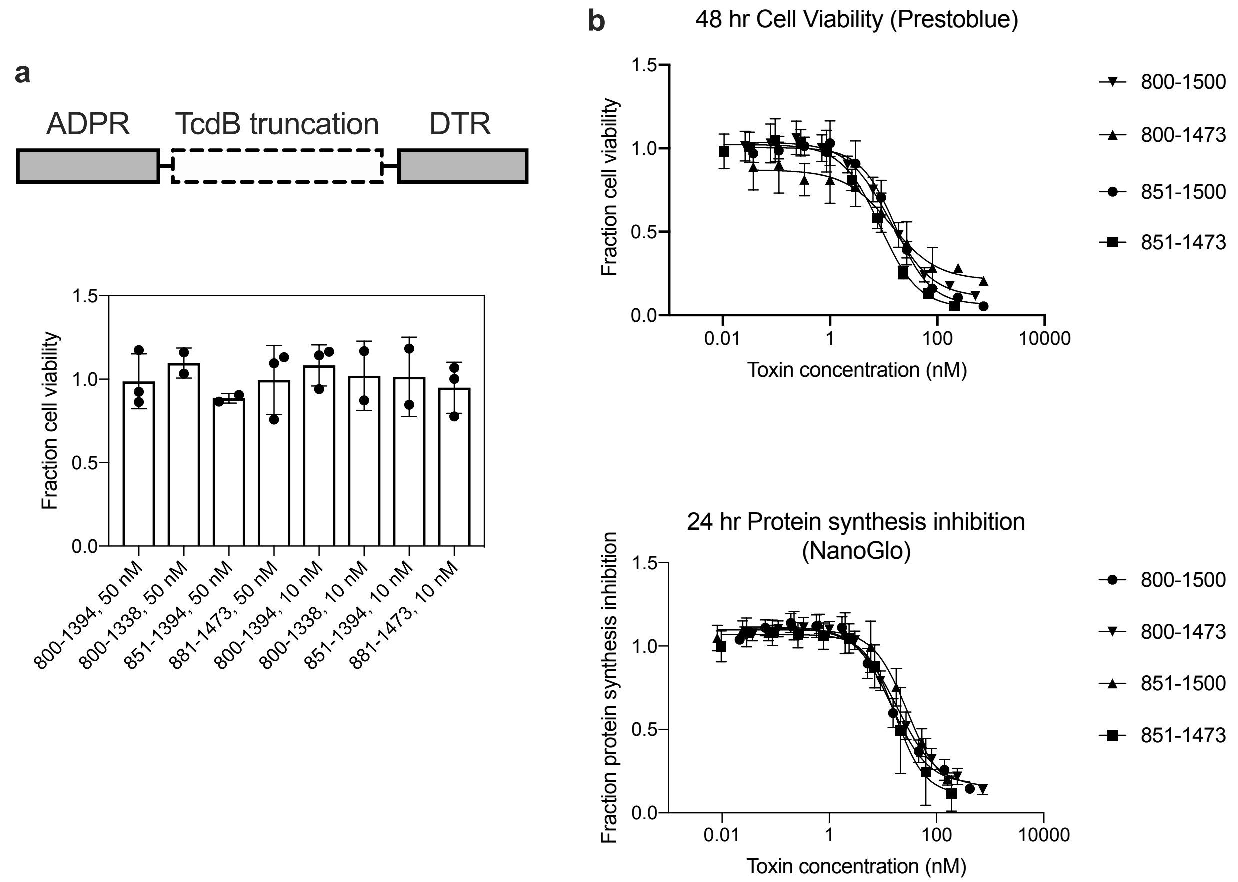

Supplementary Figure 7 Cell viability and protein synthesis inhibition of ADPR-[truncated TcdB T-domain]-DTR constructs. (**a**) Fraction cell viability of all non-toxic constructs tested at both 50 nM and 10 nM. The variable TcdB truncation is indicated on the x-axis. Experiments are N=2 or N=3 (**b**) Fraction cell viability and (**c**) fraction protein synthesis inhibition curves of toxic ADPR-[truncated TcdB T-domain]-DTR constructs. The truncated TcdB T-domain is indicated on the right-hand side of the graph. Experiments are N=4. Source data are provided as a source data file for Supp Fig. 7a.

Supplementary Table 1

Pfam_ID	Pfam_Clan	Clan_Name	Pfam_Name	Pfam_Description	Correlation
**PF12920.2**			**TcdA_TcdB_pore**	**TcdA/TcdB pore forming domain**	**1**
**PF12919.2**	**CL0110**	**GT-A**	**TcdA_TcdB**	**TcdA/TcdB catalytic glycosyltransferase domain**	**0.5069**
PF03538.9			VRP1	Salmonella virulence plasmid 28.1kDa A protein	0.4184
PF06958.7	CL0446	Bacteriocin_TLN	Pyocin_S	S-type Pyocin	0.4079
PF14564.1			Membrane_bind	Membrane binding	0.3456
PF03245.8	CL0331	EpsM	Phage_lysis	Bacteriophage Rz lysis protein	0.3455
PF02413.12	CL0348	Phage_tail	Caudo_TAP	Caudovirales tail fibre assembly protein, lambda gpK	0.3453
PF07865.6			DUF1652	Protein of unknown function (DUF1652)	0.3333
PF13503.1			DUF4123	Domain of unknown function (DUF4123)	0.3283
PF12255.3			TcdB_toxin_midC	Insecticide toxin TcdB middle/C-terminal region	0.3251
PF09000.5			Cytotoxic	Cytotoxic	0.3225
PF07119.7			DUF1375	Protein of unknown function (DUF1375)	0.3104
PF11462.3	CL0266	PH	DUF3203	Protein of unknown function (DUF3203)	0.3071
PF09909.4			DUF2138	Uncharacterized protein conserved in bacteria (DUF2138)	0.3069
PF06649.7			DUF1161	Protein of unknown function (DUF1161)	0.3017
PF10109.4	CL0567	Phage_TACs	Phage_TAC_7	Phage tail assembly chaperone proteins, E, or 41 or 14	0.2971
PF09634.5			DUF2025	Protein of unknown function (DUF2025)	0.2943
PF07395.6	CL0257	Acetyltrans	Mig-14	Mig-14	0.294
PF05954.6	CL0504	Phage_barrel	Phage_GPD	Phage late control gene D protein (GPD)	0.2928
PF09498.5			DUF2388	Protein of unknown function (DUF2388)	0.2873
PF03406.8			Phage_fiber_2	Phage tail fibre repeat	0.284
PF04676.9			CwfJ_C_2	Protein similar to CwfJ C-terminus 2	0.2834
PF04717.7			Phage_base_V	Type VI secretion system, phage-baseplate injector	0.2829
PF04958.7	CL0257	Acetyltrans	AstA	Arginine N-succinyltransferase beta subunit	0.2824
PF09906.4			DUF2135	Uncharacterized protein conserved in bacteria (DUF2135)	0.2821
PF03513.9			Cloacin_immun	Cloacin immunity protein	0.2814
PF06474.7	CL0421	LppaM	MLTD_N	MltD lipid attachment motif	0.2813
**PF11647.3**			**MLD**	**Membrane Localization Domain**	**0.2767**
PF03502.8	CL0193	MBB	Channel_Tsx	Nucleoside-specific channel-forming protein, Tsx	0.2736
PF12306.3	CL0026	CU_oxidase	PixA	Inclusion body protein	0.2722
PF01320.13			Colicin_Pyocin	Colicin immunity protein / pyocin immunity protein	0.2718
PF10062.4			DUF2300	Predicted secreted protein (DUF2300)	0.2691
PF12021.3			DUF3509	Protein of unknown function (DUF3509)	0.2658
**PF11713.3**	**CL0093**	**Peptidase_CD**	**Peptidase_C80**	**Peptidase C80 family**	**0.2654**
PF08682.5	CL0236	PDDEXK	DUF1780	Putative endonuclease, protein of unknown function (DUF1780)	0.2643
PF10976.3			DUF2790	Protein of unknown function (DUF2790)	0.2637
PF06611.7			DUF1145	Protein of unknown function (DUF1145)	0.2609
PF05581.7	NA	NA	NA	NA	0.2608
PF07634.6			RtxA	RtxA repeat	0.26
PF03543.9	CL0125	Peptidase_CA	Peptidase_C58	Yersinia/Haemophilus virulence surface antigen	0.2596
PF10144.4	CL0165	Cache	SMP_2	Bacterial virulence factor haemolysin	0.258
PF13652.1			QSregVF	Putative quorum-sensing-regulated virulence factor	0.2554
PF05488.8			PAAR_motif	PAAR motif	0.2529
PF12571.3			DUF3751	Phage tail-collar fibre protein	0.2519
PF03873.8			RseA_C	Anti sigma-E protein RseA, C-terminal domain	0.2517
PF06790.6			UPF0259	Uncharacterised protein family (UPF0259)	0.2471
PF05736.6	CL0193	MBB	OprF	OprF membrane domain	0.244
PF13693.1	CL0123	HTH	HTH_35	Winged helix-turn-helix DNA-binding	0.2417
PF04320.9			DUF469	Protein with unknown function (DUF469)	0.2403
PF03889.8			ArfA	Alternative ribosome-rescue factor A	0.2371
PF05638.7			T6SS_HCP	Type VI secretion system effector, Hcp	0.2363
PF11293.3			DUF3094	Protein of unknown function (DUF3094)	0.2353
PF05947.7			T6SS_TssF	Type VI secretion system, TssF	0.235
PF12633.2	CL0260	NTP_transf	Adenyl_cycl_N	Adenylate cyclase NT domain	0.2347
PF09621.5			LcrR	Type III secretion system regulator (LcrR)	0.234
PF06812.7			ImpA_N	ImpA, N-terminal, type VI secretion system	0.2339
PF06942.7	CL0420	GlpM-like	GlpM	GlpM protein	0.2329
PF01295.13			Adenylate_cycl	Adenylate cyclase, class-I	0.2327
PF06672.6	CL0125	Peptidase_CA	DUF1175	Protein of unknown function (DUF1175)	0.2325
PF04984.9			Phage_sheath_1	Phage tail sheath protein subtilisin-like domain	0.232
PF04965.9			GPW_gp25	Gene 25-like lysozyme	0.2319
PF04888.7			SseC	Secretion system effector C (SseC) like family	0.2316
PF07216.7			LcrG	LcrG protein	0.2315
PF04792.7			LcrV	V antigen (LcrV) protein	0.2312
PF09025.5	CL0646	T3SS	T3SS_needle_reg	YopR, type III needle-polymerisation regulator	0.2302
PF09477.5			Type_III_YscG	Bacterial type II secretion system chaperone protein (type_III_yscG)	0.2296
PF11286.3			DUF3087	Protein of unknown function (DUF3087)	0.2274
PF06693.6			DUF1190	Protein of unknown function (DUF1190)	0.2271
PF09619.5			YscW	Type III secretion system lipoprotein chaperone (YscW)	0.2258
PF03573.8	CL0193	MBB	OprD	outer membrane porin, OprD family	0.2242
PF11862.3			DUF3382	Domain of unknown function (DUF3382)	0.2198
PF05844.7			YopD	YopD protein	0.2175
PF10948.3			DUF2635	Protein of unknown function (DUF2635)	0.2161
PF11661.3			DUF2986	Protein of unknown function (DUF2986)	0.2158
PF06450.7	CL0182	IT	NhaB	Bacterial Na+/H+ antiporter B (NhaB)	0.2158
PF07023.7			DUF1315	Protein of unknown function (DUF1315)	0.2149
PF08468.6			MTS_N	Methyltransferase small domain N-terminal	0.2149
PF07409.7			GP46	Phage protein GP46	0.2135
PF14567.1	CL0526	SUKH	SUKH_5	SMI1-KNR4 cell-wall	0.2133
PF09904.4	CL0123	HTH	HTH_43	Winged helix-turn helix	0.2132
PF05597.6			Phasin	Poly(hydroxyalcanoate) granule associated protein (phasin)	0.2131
PF10618.4			Tail_tube	Phage tail tube protein	0.2125
PF09392.5			T3SS_needle_F	Type III secretion needle MxiH, YscF, SsaG, EprI, PscF, EscF	0.2116
PF12790.2	CL0287	Transthyretin	T6SS-SciN	Type VI secretion lipoprotein, VasD, EvfM, TssJ, VC_A0113	0.2116
PF05106.7	CL0564	Holin-III	Phage_holin_3_1	Phage holin family (Lysis protein S)	0.2113
PF04985.9	CL0569	Phage_TTPs	Phage_tube	Phage tail tube protein FII	0.2111
PF07201.6	CL0646	T3SS	HrpJ	HrpJ-like domain	0.2105
PF06890.7			Phage_Mu_Gp45	Bacteriophage Mu Gp45 protein	0.2103
PF05936.7			T6SS_VasE	Bacterial Type VI secretion, VC_A0110, EvfL, ImpJ, VasE	0.2103
PF06476.7			DUF1090	Protein of unknown function (DUF1090)	0.2097
PF06295.7			DUF1043	Protein of unknown function (DUF1043)	0.2097
PF11340.3			DUF3142	Protein of unknown function (DUF3142)	0.2096
PF06995.6			Phage_P2_GpU	Phage P2 GpU	0.2096
PF11354.3			DUF3156	Protein of unknown function (DUF3156)	0.2096
PF10679.4			DUF2491	Protein of unknown function (DUF2491)	0.2089
PF03865.8	CL0193	MBB	ShlB	Haemolysin secretion/activation protein ShlB/FhaC/HecB	0.2089
PF03974.8			Ecotin	Ecotin	0.2084
PF06794.7			UPF0270	Uncharacterised protein family (UPF0270)	0.208
PF07157.7			DNA_circ_N	DNA circularisation protein N-terminus	0.208
PF09500.5	CL0050	HotDog	YiiD_C	Putative thioesterase (yiiD_Cterm)	0.2072

Supplementary Table 1 Proteome-wide Pfam annotations were retrieved from the GTDB. For all bacterial proteomes in the GTDB, the presence of the TcdA/B Pore-Forming domain Pfam model (PF12920) was correlated to the presence/absence of all other Pfam domains (using Pearson's *r* correlation coefficient). The top 100 correlating domains are listed below in order of decreasing correlation coefficient. Notably, the presence of the LCT glucosyltransferase and cysteine peptidase domains were among the top 100 domains correlated with the presence of the translocase domain (*r* = 0.5069 and 0.2654, respectively). Other domains within the top 100 include components of several bacterial secretion systems, as well as other toxin-related domains.

References

1 Mendler, K. *et al.* AnnoTree: visualization and exploration of a functionally annotated microbial tree of life. *Nucleic Acids Res* **47**, 4442-4448, doi:10.1093/nar/gkz246 (2019).
